# Effectiveness of direct needle puncture for complete hepaticojejunostomy anastomotic stricture after pancreaticoduodenectomy (with video)

**DOI:** 10.1002/deo2.396

**Published:** 2024-06-27

**Authors:** Koichi Soga, Fuki Hayakawa, Takeshi Fujiwara, Yoshinori Gyotoku, Yumi Kusano, Ikuhiro Kobori, Masaya Tamano

**Affiliations:** ^1^ Department of Gastroenterology Dokkyo Medical University Saitama Medical Center Saitama Japan

**Keywords:** anastomotic stricture, biliary drainage, hepaticojejunostomy, injection needle, pancreaticoduodenectomy

## Abstract

A 79‐year‐old Japanese woman, who had undergone pancreaticoduodenectomy 6 months prior to presentation owing to pancreatic cancer, complained of jaundice with high fever. Computed tomography revealed proximal bile duct dilatation with complete hepaticojejunostomy anastomotic stricture (HJAS). We performed a single‐balloon endoscopy for biliary drainage. The presence of a scar‐like feature surrounding the anastomosis was identified as the HJAS. White‐light imaging during single‐balloon endoscopy revealed that the HJAS contained a milky whitish area (MWA), suggesting that a membranous and fibrosis layer affected continuous inflammation around the center of the anastomosis (within a scar‐like feature). Endoscopic dilatation was performed using an endoscopic injection needle, with the MWA used as an indicator. A 23‐gauge endoscopic injection needle was used to penetrate the center of the blind lumen within the MWA, and a pinhole was created in the stricture. After confirming the position of the proximal bile duct using a contrast medium with the needle, an endoscopic guidewire with a cannula was inserted into the pinhole. A through‐the‐scope sequential balloon dilator was used to dilate the stricture, and a plastic stent was inserted into the proximal bile duct. This endoscopic intervention led to positive outcomes. In cases of complete HJAS occlusion, an endoscopic approach to the bile duct is difficult because the anastomotic opening of the HJAS is not visible. Thus, puncturing within the MWA, which can be used as a scar‐like landmark within a complete membranous HJAS, is considered a useful endoscopic strategy.

## INTRODUCTION

Pancreatobiliary endoscopic interventions using balloon‐assisted endoscopes are the first‐line therapy for pancreatobiliary diseases in postoperative patients with reconstructed gastrointestinal anatomy.^1^ However, there are many technical difficulties, and procedural completion rates vary widely among institutions, indicating that procedural techniques are yet to be standardized.

In patients with hepaticojejunostomy anastomotic stricture (HJAS), recurrent cholangitis and jaundice may occur several months to years postoperatively. Longer survival after pancreaticoduodenectomy (PD) is associated with a higher incidence of benign HJAS.^2^ HJAS causes liver injury, obstructive jaundice, and cholangitis, all requiring treatment. In cases of complete HJAS/occlusion, an endoscopic approach to the bile duct is difficult because the anastomotic opening of the HJAS is not visible. Here, we describe a patient who underwent direct needle puncture for complete HJAS after PD.

## CASE REPORT

**FIGURE 1:**
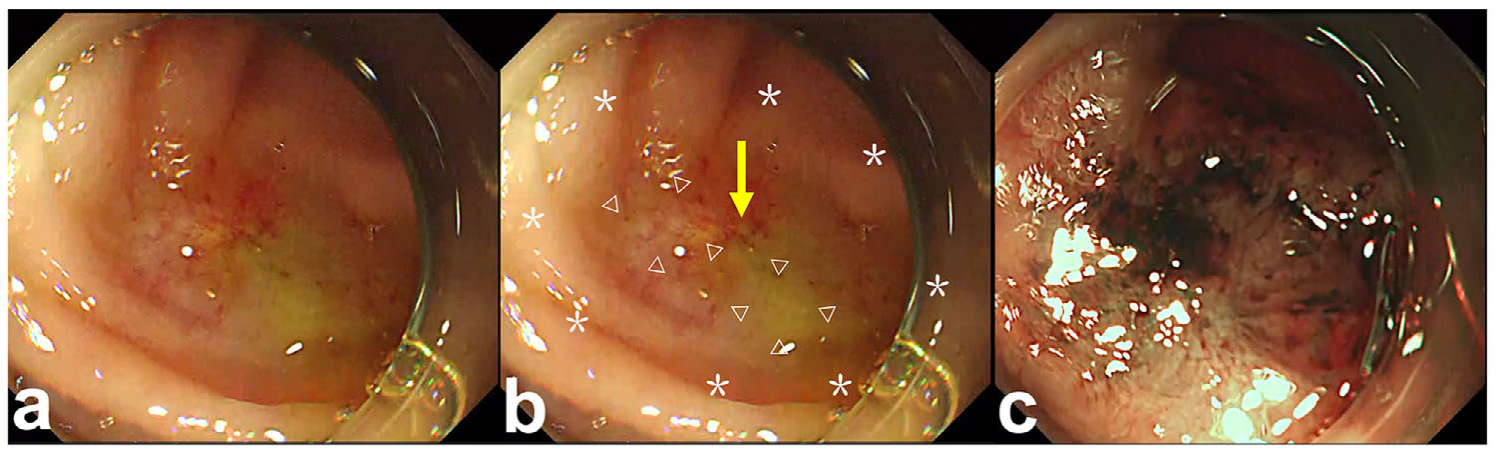
Course of endoscopic dilatation for complete hepaticojejunostomy anastomotic stricture. Single‐balloon endoscopy for biliary drainage. The presence of an ulcer scar‐like mucosa surrounding the anastomosis is identified as the hepaticojejunostomy anastomotic stricture (a). White‐light imaging during single‐balloon endoscopy reveals a milky whitish area (b: triangle) present within the hepaticojejunostomy anastomotic stricture (b: asterisk), suggesting that a membranous and fibrosis layer affected continuous inflammation around the center of the anastomosis (within a scar‐like feature). The endoscopic narrow band image clearly shows the mucosal concentration toward the center of the anastomosis, which enables a detailed evaluation of the stenotic area (c).

A 79‐year‐old Japanese woman, who had undergone PD 6 months previously for pancreatic cancer, complained of jaundice with a high fever. Computed tomography revealed a proximal bile duct dilatation with HJAS. We performed a single‐balloon endoscopy (SBE) for biliary drainage. The presence of an ulcer scar‐like mucosa surrounding the anastomosis was identified as the HJAS. White‐light imaging during SBE revealed that a milky whitish area (MWA) was present within the HJAS, suggesting that a membranous and fibrosis layer affected continuous inflammation around the center of the anastomosis (within the scar‐like feature). The endoscopic narrow band image clearly showed the mucosal concentration toward the center of the anastomosis, which enabled a detailed evaluation of the stenotic area (Figure 1). We attempted to perform biliary drainage using an endoscopic device.

**FIGURE 2:**
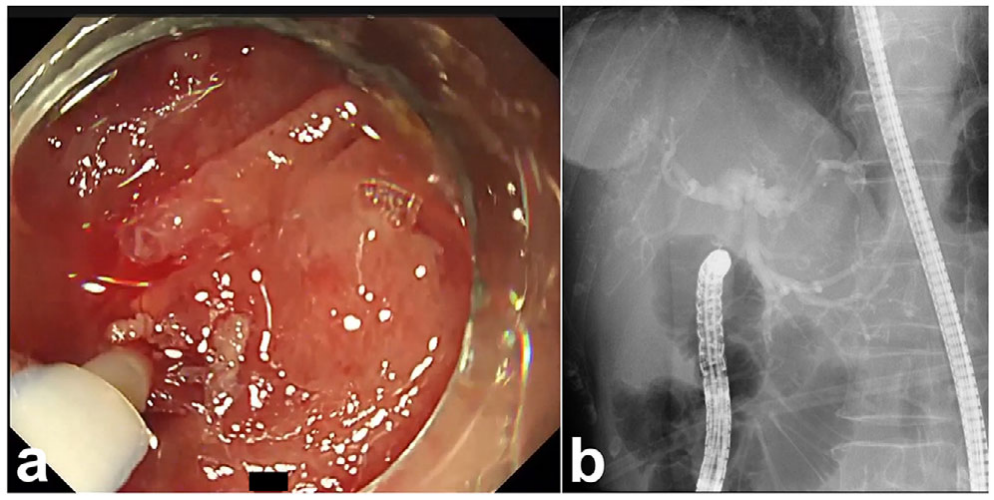
Hepaticojejunostomy anastomotic stricture puncture using a 23‐G endoscopic injection needle. A 23‐G endoscopic injection needle is used to penetrate the center of the blind lumen supporting the use of the milky whitish area as an indicator, and a pinhole is created in the stricture (a). The position of the proximal bile duct is confirmed using a contrast medium (b).

**FIGURE 3:**
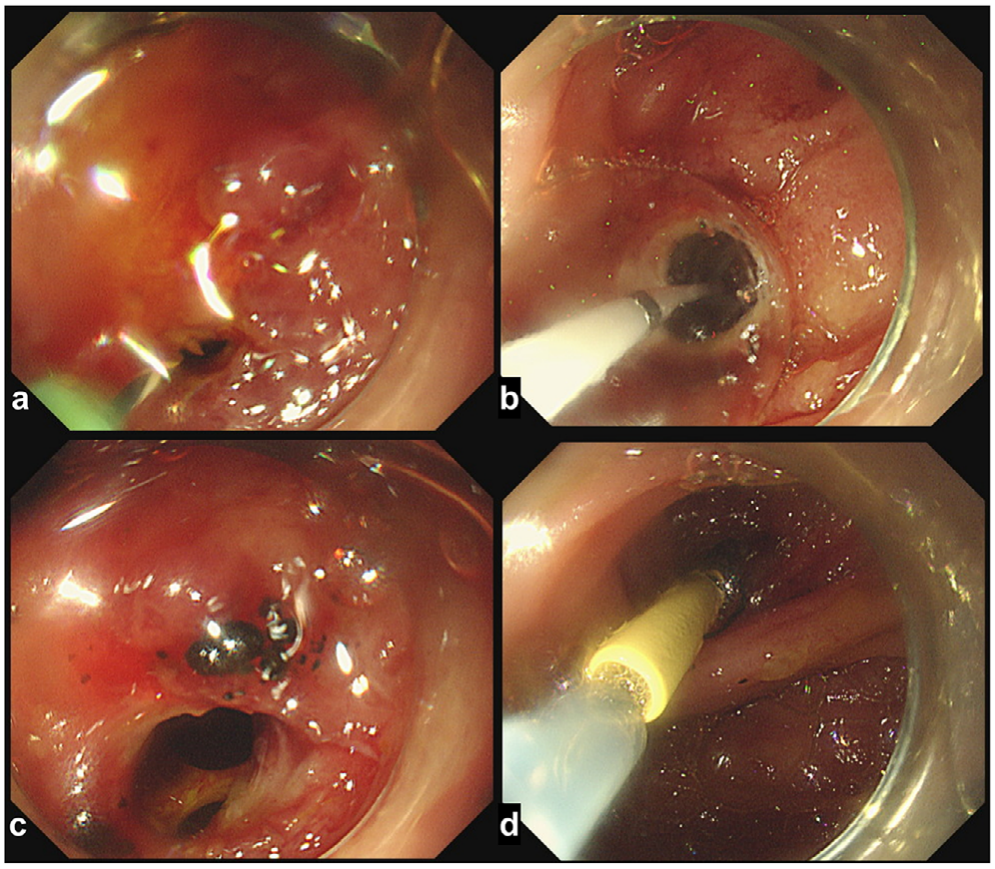
Dilatation of the hepaticojejunostomy anastomotic stricture. After the needle puncture, an endoscopic guidewire is inserted into the pinhole (a). A through‐the‐scope sequential balloon dilator (8 mm, REN; Kaneka) is used to dilate the stricture (b). We have confirmed that the hepaticojejunostomy anastomotic stricture is endoscopically dilated, and the stenosis has improved endoscopically (c). Subsequently, a plastic stent (7 French × 9 cm; Through & Pass; Gadelius) is inserted into the proximal bile duct (d).

**FIGURE 4:**
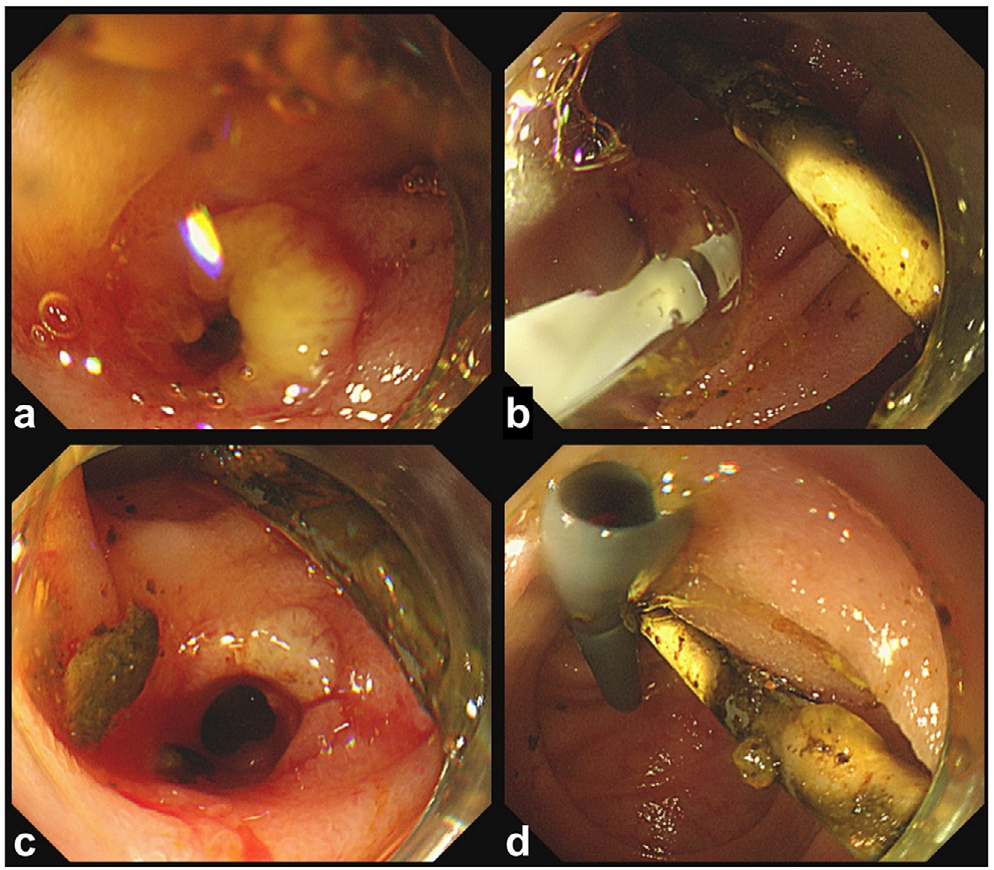
Four months after the initial endoscopic dilatation, the patient underwent stent replacement owing to stent dysfunction. The lumen of the anastomosis is fully open when the plastic stent is removed (a). A through‐the‐scope sequential balloon dilator (8 mm, REN; Kaneka) is used to dilate the stricture (b, c), and then a plastic stent (8.5 French × 10 cm, SUZAKU; Kaneka) is inserted into the proximal bile duct (d).

To drain the bile juice from the HJAS, we first attempted to intubate the supposed bile duct orifice with an endoscopic retrograde cholangiopancreatography cannula and guidewire, but this was not possible. Then, a 23‐gauge (G) endoscopic injection needle (total length, 2200 mm; needle length, 3 mm; Top Corp.) was used to penetrate the blind lumen within the MWA, and a pinhole was created in the stricture. After confirming the position of the proximal bile duct using a contrast medium with the needle (Figure 2), an endoscopic cannula and guidewire were inserted into the pinhole. A through‐the‐scope sequential balloon dilator (8 mm, REN; Kaneka) was used to dilate the stricture. We confirmed that the HJAS was endoscopically dilated and that the stenosis had improved. A plastic stent (7 French × 9 cm; Through & Pass; Gadelius) was inserted into the proximal bile duct (Figure 3; Video S1). This endoscopic intervention led to positive outcomes. Four months after the initial endoscopic dilatation, the patient underwent stent replacement because of stent dysfunction as the stenosis at the bile duct jejunal anastomosis improved (Figure 4).

## DISCUSSION

Postoperative biliary stricture is a rare but serious complication of biliary surgery. These recurrent strictures can be challenging to manage. Herein, we performed a detailed evaluation of complete HJAS after radical PD using SBE. The stricture was subsequently opened by puncturing the site with an injection needle, followed by balloon dilatation for complete drainage.

Despite improvements in technology and surgical techniques, the incidence of HJAS complications remains stable and substantial, and detecting and treating anastomotic complications remains challenging. Benign anastomotic stenosis is a late complication of HJAS. The pathogenesis of HJAS after PD is multifactorial. Five main factors are responsible for the development of HJAS: tension of the anastomosis, ischemia of the anastomosis, suturing technique, reflex cholangitis with bile juice reflux to the remnant bile duct, and leakage of the anastomosis.^3^ This process is a normal part of the post‐surgical tissue response; yet, excessive scar formation may cause pathological stricture.

In complete HJAS, it is difficult to find the anastomosis because the opening cannot be identified, and there is little bile outflow into the jejunum. The presence of a scar‐like mucosa surrounding the anastomosis can also serve as a clue for finding the anastomotic site.^1^

We would like to discuss two points: (a) evaluation of the stenosis and (b) evaluation of the endoscopic procedure. First, the critical point of focus involves identifying the HJAS and determining the precise site for puncture. Shimatani et al. discussed the importance of targeting a scar‐like feature for puncture. They deliberated on the formation of ulcerative scar‐like mucosa at the postoperative HJAS, which is typically due to a fibrotic reaction that causes constriction, with the mucosa becoming scarred and mimicking the appearance of an ulcer. Managing such conditions requires precise identification of the stricture and sophisticated endoscopic techniques to administer effective treatment.^1^

Furthermore, ensuring that the procedure progresses without complications following the needle puncture is vital. The risk of unsuccessful outcomes arises from potential blind punctures, external biliary punctures, or inadequate punctures. Thus, reliably identifying the scar‐like feature, pinpointing the safest possible puncture sites within it, and executing the puncture can substantially enhance the success rate. We consider that the MWA reported herein is a helpful marker for assessing HJAS. One might speculate that the MWA may be the result of an immune response to inflammation by macrophages, fibroblast induction, and phagocytic activity.^4^

Second, several techniques for improving occlusion have been reported, including the rendezvous technique combining percutaneous cholangioscopy,^5^, ^6^ interventions using endoscopic ultrasonography,^7^ recanalization by magnetic compression anastomosis,^8^ and biliary puncture cholangiography using an injection needle.^9^ Here, HJAS punctures were performed using a 23‐G injection needle. Raitel et al. reported the classification of benign HJAS to be valuable, categorizing them into three types: mucosal, intramural, and ductal. Challenging endoscopic cases of HJAS often involve mucosal, intramural, and combinations of mucosal and intramural types. Based on Raitel et al.’s study, the mucosal type of HJAS measures approximately 2.6 ± 0.8 mm; thus, considering our use of a 3‐mm injection needle, it is feasible to perform the puncture from the jejunum into the bile duct without issues.^10^

Generally, injection needles are used in endoscopic mucosal resection or endoscopic submucosal dissection for submucosal injections. The injection needle is 3–4 mm long and 23–26 G thick. Thus, no larger‐diameter injection needles can pass through a 0.018‐ and 0.025‐inch guidewire in the Japanese market and are compatible in length with SBE. Moreover, the need for a larger‐diameter injection needle is becoming less necessary because of advances in the use of smaller needles to inject local injection fluids for safety reasons.

For this reason, we attempted to relieve the complete stenosis with an injection needle by puncture and contrast with a 23‐G injection needle. However, a blind HJAS puncture is problematic. It was not possible to determine with certainty how far the tip of the needle had been inserted. Therefore, there is a risk of injection through or out of the bile duct if a deep puncture is performed. To minimize this, we identified the area most like an MWA and punctured this area minimally without deep penetration as a guidewire insertion marker. Other preoperative modalities, such as computed tomography, magnetic resonance imaging, and ultrasonography, must be performed to ensure no tumor changes and assess the diameter of bile duct dilatation.

The MWA was identified within the complete HJAS, and a needle was punctured within the MWA as a supporting marker, minimizing the risk of blind puncture. Methodology and this technique‐specific device development are desirable to ensure the safety of this technique in the future.

In cases of complete HJAS occlusion, an endoscopic approach to the bile duct is difficult because the anastomotic opening of the HJAS is not visible. An endoscopic approach involving a puncture as a supporting marker within the MWA for complete HJAS was found to be a useful technique.

## CONFLICT OF INTEREST STATEMENT

None.

## PATIENT CONSENT STATEMENT

Obtained.

## ETHICAL STATEMENT

None.

## Supporting information

VIDEO S1 Effectiveness of direct needle puncture for complete hepaticojejunostomy anastomotic stricture (HJAS) after pancreaticoduodenectomy (PD).
